# Determinants of Use of Intermittent Preventive Treatment of Malaria in Pregnancy: Jinja, Uganda

**DOI:** 10.1371/journal.pone.0015066

**Published:** 2010-11-29

**Authors:** Laura R. Sangaré, Andy Stergachis, Paula E. Brentlinger, Barbra A. Richardson, Sarah G. Staedke, Mpungu S. Kiwuwa, Noel S. Weiss

**Affiliations:** 1 Department of Global Health, University of Washington, Seattle, Washington, United States of America; 2 Department of Epidemiology, University of Washington, Seattle, Washington, United States of America; 3 Department of Biostatistics, University of Washington, Seattle, Washington, United States of America; 4 Department of Clinical Research, London School of Hygiene and Tropical Medicine, London, United Kingdom; 5 Infectious Disease Research Collaboration, Kampala, Uganda; 6 Clinical Epidemiology, School of Medicine, College of Health Sciences, Makerere University, Kampala, Uganda; Université Pierre et Marie Curie, France

## Abstract

**Background:**

Maternal malaria is associated with serious adverse pregnancy outcomes. One recommended means of preventing malaria during pregnancy is intermittent preventive therapy (IPTp) with sulfadoxine/pyrimethamine (SP). We sought to identify determinants of preventive use of SP during pregnancy among recently pregnant women in Uganda. Additionally, we characterized the timing of and indications for the administration of SP at antenatal care (ANC) visits and missed opportunities for SP administration.

**Methodology/Principal Findings:**

Utilizing a population-based random sample, we interviewed 500 women living in Jinja, Uganda who had been pregnant in the past year. Thirty-eight percent (192/500) of women received SP for the treatment of malaria and were excluded from the analysis of IPTp-SP. Of the remaining women, 275 (89.3%) reported at least two ANC visits after the first trimester and had an opportunity to receive IPTp-SP according to the Ugandan guidelines, but only 86 (31.3%) of these women received a full two-dose course of IPTp. The remaining 189 (68.7%) women missed one or more doses of IPTp-SP. Among the 168 women that were offered IPTp, 164 (97.6%) of them took the dose of SP.

**Conclusions/Significance:**

Use of IPTp in Uganda was found to be far below target levels. Our results suggest that women will take SP for IPTp if it is offered during an ANC visit. Missed opportunities to administer IPTp-SP during ANC were common in our study, suggesting provider-level improvements are needed.

## Introduction

Approximately 30 million pregnancies occur each year in malaria endemic areas of sub-Saharan Africa [1]. Severe complications of malaria during pregnancy include cerebral malaria, maternal anemia, and maternal mortality, which tend to be more frequent during epidemics and among primigravid and/or immunocompromised pregnant women [2], [3]. Complications affecting the fetus or newborn may arise from either clinical malaria or asymptomatic parasitemia during pregnancy and include miscarriage, stillbirth, low birthweight, preterm delivery, and neonatal mortality [2], [3].

The World Health Organization (WHO) guidelines for the prevention of malaria during pregnancy include 1) use of intermittent preventive treatment during pregnancy (IPTp) with sulfadoxine/pyrimethamine (SP); and 2) sleeping under an insecticide-treated bed net (ITN) [4]. IPTp-SP is defined as provision of treatment doses of SP to asymptomatic individuals living in malaria endemic regions, regardless of malaria parasitemia status, and the current recommendation is that at least 2 doses of SP should be administered after the first trimester during antenatal care (ANC) [5].

Use of IPTp is estimated to reduce the occurrence of low birthweight by 42%, neonatal death by 38%, placental malaria by 65%, and antenatal parasitemia by 26% [6]. Even in areas where SP monotherapy for symptomatic malaria results in up to 25% treatment failures, 2 doses of IPTp with SP continued to provide considerable benefit to HIV-negative semi-immune pregnant women [7]. Despite the effectiveness of IPTp, and the nearly universal adoption of a national IPTp policy among malaria endemic countries [8], its use remains relatively uncommon in sub-Saharan Africa. Furthermore, data on IPTp coverage from national surveys remain limited. During 2007-2008, only 9 high-burden countries had national survey data on IPTp, resulting in only 20% of pregnant women who received 2 or more doses of IPTp [8]. In Uganda 37% of women reported receiving at least one dose of SP to prevent malaria during pregnancy, and only 18 percent received two or more doses [9].

Demographic factors associated with use of IPTp have been evaluated in several studies [10], [11], [12], [13], [14], although associations with education and age have been inconsistent across studies [10], [11], [14], [15]. A lack of association between IPTp use and socio-demographic factors household wealth, knowledge of malaria, travel times to the ANC clinic and the number of ANC visits has been reported [10], [11], [12], [14], [15]. Operational barriers affecting uptake of IPTp include: 1) imprecision in the estimation of gestational age leading to missed doses [16], [17]; 2) confusion regarding the timing of doses and/or the recommended number of SP doses [16], [17], [18]; 3) late or no antenatal care attendance [16], [17], [19]; and lack of potable water and/or drinking cups (for directly observed SP administration) [16], [17], [18].

Utilizing a population-based sample, we sought to describe the use of SP during pregnancy in one health district in Uganda and to identify determinants of use of IPTp. Additionally, we sought to describe the administration practices of SP during visits to the ANC clinic.

## Methods

### Selection of study participants

Between November, 2008 and January, 2009 a simple random sample of 500 female residents of Kibibi and Namizi parishes in Budondo-sub county of Jinja District, Uganda was invited to participate in a home-based interview to ascertain use of ITNs and SP during pregnancy, as well as possible factors associated with use. Interviews were conducted using a structured pre-tested questionnaire adapted from the conceptual framework proposed by Ribera et al. [20]. Women between the ages of 15 and 49 years who had a pregnancy within the past 12 months that lasted until at least the third trimester, regardless of pregnancy outcome, were eligible to participate. Due to the cross-sectional design of the study, current pregnancies were excluded to ensure equal opportunity among all participants to have received IPTp during their most recent pregnancy. Budondo-sub county of Jinja District was selected as the field site based on the availability of a recently completed census in November 2008, allowing for a population-based simple random sample to be selected. Namizi and Kibibi parishes are comprised of 16 rural and peri-urban villages, with a combined population of 21,681, of whom 4,654 were females aged 15–49 years, and 867 of these women reported having been pregnant in the previous 12 months.

### Study site

Jinja district is a peri-urban area where malaria is considered meso-endemic, with a relatively low transmission intensity; the average annual entomological inoculation rate is 6 infective bites per person per year [21].

Each parish has one public health center; Kibibi has a level II facility and Namizi a level IV facility. The administration policy of IPTp and the frequency of stock-outs of SP at the study clinics were assessed prior to the start of the study. Stock-outs of SP during the study period were uncommon (Namizi and Kibibi health centers, personal communication). While the Ugandan guidelines specify IPTp with SP should be taken as directly observed therapy (DOT), this is not consistently implemented in the study clinics due to lack of access to clean water and cups.

### Administration of the survey

For each ANC visit the woman attended, we ascertained if SP was offered or not and categorized her experience as 1) having received SP; 2) out of stock of SP, the woman was told to buy it on her own or return to ANC later to receive it; 3) asked to buy SP from the ANC; 4) the ANC never mentioned SP, or 5) SP was offered, but the woman declined to use it.

To facilitate recall, a pregnancy history calendar was generated for each woman and used to record episodes of self-reported malaria, any use of SP or other antimalarials during pregnancy, and ANC visits. Additionally, women were shown photographs of SP packaging and the corresponding tablets for the most common formulations of SP available in the area. Self-reported SP use was compared with SP administration as recorded on antenatal cards for the subset of women who had retained the cards.

### Data management

IPTp with SP was defined as a complete 2-dose course of SP administered after the first trimester [9], [22], [23], [24], [25], if the participant believed the SP was used for the prevention of malaria. The analysis of IPTp was restricted to those participants with at least two qualifying ANC visits after the first trimester who had the opportunity to receive a complete course of IPTp. The indication for the use of SP (treatment or prevention) was based on self-report from the woman by asking her if she believed she was sick with malaria for each of the doses of SP that she received. Women who reported receiving SP for the treatment of malaria symptoms were excluded from the analysis of IPTp-SP for the following reasons: 1) determinants of use of treatment doses among women with symptoms suggestive of malaria are likely to be different than those of preventive doses among asymptomatic women; and 2) women who received a therapeutic dose of SP administered in concordance with the IPTp schedule would be unlikely to receive the recommended two or more preventive doses of SP. The Ugandan IPTp guidelines recommend that SP should not be given: during the first trimester of pregnancy, less than 4 weeks between doses, to women with a history of allergies to sulfa drugs, to women concurrently using cotrimoxazole, or to women with symptomatic malaria [26].

IPTp-SP was categorized as a *full course*: a complete 2-dose course of IPTp administered after the first trimester; *partial course*: only 1 dose after the first trimester; or *none*: a) 0 doses in the after the 1^st^ trimester.

### Statistical analysis

Analyses were performed using Stata version 11.0 (College Station, Texas, USA). A 7-point composite variable was generated to summarize each woman's knowledge of malaria, and a 4-point composite variable summarized her knowledge of SP safety. Principal components analysis was used to calculate the household wealth index, a standardized composite measure combining the cumulative living standard of a household and is based on a household's ownership of selected assets, such as televisions and bicycles, materials used for housing construction, and types of water access and sanitation facilities [27]. Relative risk regression was used to determine the association between exposures of interest and receipt of a full-course of IPTp-SP [28], [29]. Risk estimates were adjusted for the number of ANC visits, however, small numbers precluded further adjustments.

### Ethics statement

The study was approved by the Makerere University Research and Ethics Committee, the Uganda National Council for Science and Technology, and the University of Washington, Human Subjects Division. All participants provided written informed consent.

## Results

**Figure 1:**
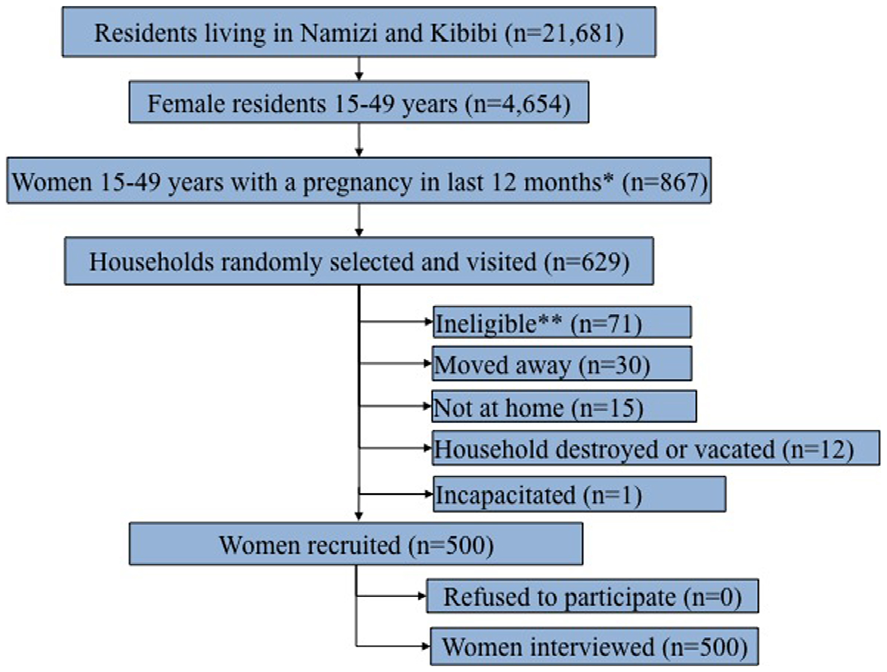
Eligibility and participation of study participants. *Reflects completed pregnancies lasting at least until the beginning of the 3^rd^ trimester, regardless of pregnancy outcome. **Most recent pregnancy occurred more than 12 months from the interview date due to a lag time between the start of the census and the start of the study.

Between November 2008 and January 2009 we visited 629 households to identify 500 eligible women (Figure 1), none of whom declined to participate. Seven of the index pregnancies ended in stillbirth. Use of ANC was nearly ubiquitous with 94.2% of women reporting 2 or more visits, and 90.8% reporting 2 or more ANC visits after the first trimester. Only 34.0% completed the recommended 4 or more visits during pregnancy. Most of the women delivered in a health facility (73.4%), and 16.2% delivered at home. Symptomatic malaria during pregnancy was self-reported by 66.8% of the participants. The characteristics of women with at least two qualifying ANC visits were similar to those of the entire cohort and are shown in Table 1.

**Table 1:** Characteristics of the study population.

Characteristic	All women interviewedn = 500	Women with at least 2 qualifying ANC visitsn = 275^1^
Age (years); mean (sd)	25.5 (6.2)	25.4 (6.3)
≤18 years	50 (10.0)	30 (10.9)
19–24 years	204 (40.8)	114 (41.4)
25–34 years	182 (36.4)	96 (34.9)
≥35 years	64 (12.8)	35 (12.7)
Married; n (%)	451 (90.2)	247 (89.8)
Education; n (%)		
None	34 (6.8)	14 (5.1)
Primary	350 (70.0)	197 (71.6)
Secondary/Postsecondary	116 (23.2)	64 (23.3)
Religion; n (%)		
Christian-based	305 (61.0)	160 (58.2)
Muslim	195 (39.0)	115 (41.8)
Village type; n (%)		
Rural	372 (74.4)	213 (77.4)
Peri-Urban	128 (25.6)	62 (22.6)
Number of births; mean (sd)	3.9 (2.6)	3.8 (2.6)
Knowledge of malaria score; n (%)		
High	292 (58.4)	162 (58.9)
Low	208 (41.6)	113 (41.1)
Belief SP is safe during pregnancy; n (%)	248 (49.7)	122 (44.4)
Knowledge of SP safety score; n (%)		
High	447 (89.4)	240 (87.3)
Low	53 (10.6)	35 (12.7)
Who decides if SP is used during pregnancy? n (%)		
Respondent	159 (31.8)	90 (32.7)
Husband/partner	25 (5.0)	15 (5.4)
Respondent and husband jointly	130 (26.0)	84 (30.6)
Someone else	186 (37.2)	86 (31.3)
Most important influence to use SP; n (%)		
Given free at ANC	97 (19.4)	53 (19.3)
Recommended by a doctor or nurse	341 (68.2)	193 (70.2)
Ad on radio or poster	23 (4.6)	12 (4.4)
Hearing from other pregnant women	39 (7.8)	17 (6.2)

1 (n = 275): Excludes women without 2 qualifying ANC visits in the 2^nd^ and 3^rd^ trimester (n = 46), women using SP for the treatment of malaria (n = 179).

### Concordance of self-reported SP and ANC cards

Twenty-seven percent of women (136/500) had an ANC card available. The number of SP doses used for any reason (treatment or prevention) was listed on 106 of the cards and ranged from 0 to 4 doses. The concordance between self-reported SP doses and ANC card was high (Pearson's rho  = 0.93).

Reasons for SP use among women who had an ANC card available were as follows: prevention only 40.1%, any treatment use 36.8%, or no use 22.6%. Excluding women who received SP for treatment, the concordance between self-reported SP doses and ANC card remained high (Pearson's rho  = 0.95).

### Any SP use during pregnancy

Seventy-three percent of participants (365/500) reported having taken a dose of SP at least once during pregnancy for prevention or treatment of malaria; 28.2% (n = 141) reported taking 1 dose and 44.8% (n = 224) reported taking 2 or more doses. Among SP users, 173 (47.4%) used SP for prevention only (believed they did not have symptomatic malaria each time they took SP), 132 (36.2%) used SP for treatment only (believed they did have symptomatic malaria each time they took SP), and the remaining 60 (16.4%) used SP as both treatment and prevention.

### Administration of SP by the health facility

Among all participants, 90.8% (n = 454) of them had at least two ANC visits in which a full course of IPT-SP could have been administered according to the Ugandan guidelines. However, SP was not administered in accordance with recommendations in 81.1% (n = 368) of these women: 41.6% (n = 189) missed an opportunity to receive the recommended 2 preventive doses during pregnancy (0 doses: n = 110; 1 dose: n = 79), and 39.4% (n = 179) believed they received SP as treatment for an episode of malaria during pregnancy.

### IPTp-SP use during pregnancy among women with 2 or more qualifying ANC visits

While the proportion of women who received 2 or more doses of SP was 44.8% (224/500), only 31.3% (86/275) of women received a full 2-dose course of IPTp-SP according to the Ugandan policy for the prevention of malaria (Table 2). A partial course of IPTp was used among 28.7% of women. The number of women receiving no doses of SP in the 2^nd^ and/or 3^rd^ trimesters despite qualifying ANC visits was 40.0%.

**Table 2:** Doses of IPTp-SP taken during pregnancy among women with 2 or more qualifying ANC visits.

Number of doses of SP-IPT^1^	n (%)
*Full course*	86 (31.3)
≥2 doses in 2^nd^ AND 3^rd^ trimesters	64
≥2 doses in 2^nd^ or 3^rd^ trimesters	22
*Partial course*	79 (28.7)
<2 doses in 2^nd^ or 3^rd^ trimesters, no incorrect doses	73
<2 doses in 2^nd^ or 3^rd^ trimesters, plus 1^st^ trimester use	6
*None*	110 (40.0)
0 doses	109
1^st^ trimester use only	1

1 (n = 275): Excludes women without qualifying ANC visits in the 2^nd^ and 3^rd^ trimester (n = 46), women using SP for the treatment of malaria (n = 179).

Among the 168 women that were offered IPTp, 164 (97.6%) of them took the dose of SP. Only 2 women used SP for prevention despite never being offered it during an ANC visit, and only 4 women who were offered SP for prevention during an ANC visit chose not to use it.

### Predictors of IPTp-SP among women with 2 or more qualifying ANC visits

Individual-level factors associated with receipt of a full-course of IPTp-SP compared to no doses were assessed among women with at least 2 qualifying ANC visits controlling for the total number of ANC visits (Table 3). Receipt of a full-course of IPTp-SP was relatively more common among women living in a rural village compared to a peri-urban area (RR: 2.73; 95% CI: 1.50, 4.99), those with the capacity to decide if SP should be used during pregnancy (RR: 2.28; 95% CI: 1.48, 3.49), and those who were less knowledgeable about the safety of SP use during pregnancy (RR: 1.87; 95% CI: 1.40, 2.49). Furthermore, women with lower educational attainment were more likely to receive a full-course (RR: 1.56; 95% CI: 1.03, 2.38), as were women living more than 30 minutes walking distance to the ANC clinic (RR: 2.06; 95% CI: 1.23, 3.46). No differences were found between other socio-demographic factors, pregnancy history, or socio-cultural factors.

**Table 3:** Individual-level factors associated with full adherence to IPTp-SP recommendations among women with at least 2 qualifying ANC visits.

Characteristic	Full coursen = 86	No dosesn = 110	aRR (95% CI)
Age (years); n (%)			
≤18 years	10 (41.7)	14 (58.3)	0.77 (0.47, 1.26)
19–24 years	39 (52.7)	35 (47.3)	Reference
25–34 years	25 (36.8)	43 (63.2)	0.74 (0.51, 1.08)
≥35 years	12 (40.0)	18 (60.0)	0.76 (0.46, 1.24)
Marital status; n (%)			
Single	8 (36.4)	14 (63.6)	0.97 (0.55, 1.70)
Married	78 (44.8)	96 (55.2)	Reference
Education; n (%)			
None	2 (22.2)	7 (77.8)	0.79 (0.22, 2.85)
Primary	68 (49.6)	69 (50.4)	1.56 (1.03, 2.38)
Secondary/Postsecondary	16 (32.0)	34 (68.0)	Reference
Religion; n (%)			
Christian	53 (43.4)	69 (56.6)	Reference
Muslim	33 (44.6)	41 (55.4)	1.04 (0.76, 1.45)
Parish; n (%)			
Kibibi	34 (41.5)	48 (58.5)	Reference
Namizi	52 (45.6)	62 (54.4)	1.07 (0.78, 1.47)
Village type; n (%)			
Rural	77 (51.7)	72 (48.3)	2.73 (1.50, 4.99)
Peri-Urban	9 (19.1)	38 (80.9)	Reference
Household wealth index; n (%)			
1 (Most poor)	23 (57.5)	17 (42.5)	1.25 (0.78, 2.00)
2	13 (38.2)	21 (61.8)	0.85 (0.47, 1.53)
3	20 (45.4)	24 (54.6)	0.90 (0.53, 1.51)
4	14 (31.1)	31 (68.9)	0.67 (0.37, 1.21)
5 (Least poor)	13 (46.4)	15 (53.6)	Reference
Number of births; n (%)			
1	19 (43.2)	25 (56.8)	1.28 (0.78, 2.09)
2–3	32 (54.2)	27 (45.8)	1.50 (0.96, 2.32)
4–5	17 (42.5)	23 (57.5)	1.25 (0.74, 2.09)
≥6	18 (34.0)	35 (66.0)	Reference
Knowledge of malaria score; n (%)			
High	47 (43.9)	60 (56.1)	1.11 (0.81 1.53)
Low	39 (43.8)	50 (56.2)	Reference)
Knowledge of SP safety score; n (%)			
High	69 (39.9)	104 (60.1)	Reference
Low	17 (73.9)	6 (26.1)	1.87 (1.40, 2.49)
Who decides if SP should be used during pregnancy? n (%)			
Respondent or Respondent and husband jointly	68 (55.7)	54 (44.3)	2.28 (1.48, 3.49)
Husband/partner or someone else	18 (24.3)	56 (75.7)	Reference
Who controls money for healthcare in your household			
Respondent and husband jointly	37 (44.0)	47 (56.0)	1.02 (0.74, 1.39)
Husband/partner or someone else	49 (43.7)	63 (56.3)	Reference
Average time to walk to ANC (minutes); n (%)			
≤30	11 (24.4)	34 (75.6)	Reference
≥30	75 (49.7)	76 (50.3)	2.06 (1.23, 3.45)
Average time to wait for ANC (minutes); n (%)			
≤30	37 (45.1)	45 (54.9)	Reference
>30	49 (43.0)	65 (57.0)	1.08 (0.79, 1.47)

aRR: Adjusted for total number of ANC visits.

This analysis excludes any women who used SP for treatment of malaria.

Individual-level factors associated with receipt of a full-course compared to a partial-course of IPTp-SP were assessed among women with at least 2 qualifying ANC visits controlling for the total number of ANC visits. Women receiving a full-course of IPTp-SP had slightly less knowledge of malaria and SP compared to women receiving a partial-course (data not shown). No differences were found between these groups and the remaining socio-demographic factors, pregnancy history, and socio-cultural factors (data not shown). Among women who received a partial course of IPTp-SP, the main reasons given for not having taken a full-course of IPTp were “I didn't know about it” (49.3%), and “it wasn't offered” (34.7%).

## Discussion

In the present study of 500 recently pregnant women in Jinja, Uganda, less than one-third of women received a full-course of IPTp-SP despite the high utilization of ANC in this cohort. While IPTp is a relatively simple intervention to administer, missed opportunities were common in our study. This is one of only a few studies to investigate correlates of IPTp use from the perspective of the user.

Our definition of IPTp-SP was restricted to preventive doses of SP administered after the first trimester of pregnancy, in accordance with the IPTp policy guidelines. Including treatment doses of SP taken after the first trimester in the analysis of IPTp would lead to an overestimate of the prevalence of IPTp due to the large proportion of women receiving SP for the treatment of malaria. The means of assessment of uptake of IPTp has varied across studies and disaggregation by presence or absence of symptomatic malaria has not been standard practice. Some studies explicitly stated that preventive doses of SP were presumptive (when the woman was not sick with malaria) [10], [30], [31], [32], although timing of doses were not specified. Other studies referred only to the number of doses of SP without reference to the womans' clinical state [11], [14], [33], [34], [35]. One study defined IPTp based on number of doses of SP given at ANC irrespective of signs or symptoms of malaria [18]. Appropriate IPTp has been defined in one study based on the timing of SP use during pregnancy (at least one dose in the 2^nd^ trimester, and at least one dose in the 3^rd^ trimester), although this study did not indicate if doses were restricted to preventive use [12], while another study defined IPTp based on 3 presumptive doses starting after 16 gestational weeks, with subsequent doses being indicated at intervals of 1 month or more [16]. Surveys such as the DHS ask, “During this pregnancy, did you take any drugs to keep you from getting malaria?”, and only if the women answer “yes” will they specifically be asked if they took SP, and the number of times. Standardizing the approach to defining IPTp with SP, or other drugs, would facilitate measurement of utilization across studies.

Among women in our study, the predominant factor predicting preventive use of SP during pregnancy was being offered IPTp during an ANC visit. Stock-outs of SP were seldom responsible for missed doses; only during 5.6% of ANC visits (61/1084) was a woman told that the clinic was out of stock of SP and they should purchase it on their own. The majority of women were either offered SP and used it, or SP was never mentioned during their visit and they did not use it. Small numbers precluded an assessment of individual-level factors associated with use of IPTp independent of being offered SP for prevention. Only 2 women used SP for prevention despite never being offered it during an ANC visit, and only 4 women who were offered SP for prevention during an ANC visit chose not to use it. Our results suggest that use of IPTp was low among women in our study because providers failed to offer SP. These findings highlight the importance that health workers play in delivering IPTp and the need to explore barriers to offering this therapy.

To our knowledge, this variable “being offered” has only been assessed in two studies, both of which reported not being offered SP during ANC was the reason for not completing a full-course of IPTp-SP [15], [36]. Confusion among health care workers regarding the timing of doses and/or the number of doses has been reported in several studies, and may be the underlying reason a dose of SP was not offered during a qualifying visit [16], [17], [18]. Improvements in the administration of IPTp following focused training of health care workers was demonstrated in one intervention study [18] supporting the notion that unclear guidelines are adversely contributing to the uptake of IPTp. Further uncertainty regarding administration of IPTp may also be related to an omission in the WHO IPTp guidelines regarding how treatment for symptomatic malaria (either presumptively treated or laboratory confirmed) might alter eligibility for or timing of the IPTp. The majority of women in our study had complex histories related to self-reported malaria, and use of antimalarials for treatment of malaria was high during the index pregnancy. This led to difficulties in classifying a woman's SP use during pregnancy as being consistent or inconsistent with the recommended guidelines. For example, if a woman is sick with malaria-like symptoms and receives SP as a treatment dose in the 2^nd^ or 3^rd^ trimester, the guidelines did not specify if this dose should be counted as IPTp. Or, if a woman is sick with malaria-like symptoms and treated for confirmed or presumed malaria with non-SP antimalarials, the guidelines did not specify when she should receive SP as presumptive treatment.

We were unable to evaluate why health care workers offered a full- or partial-course of IPTp-SP to some women, and failed to offer IPTp-SP to forty percent of the remaining participants with at least two qualifying visits. While we were able to explore individual-level predictors for having received a full-course of IPTp-SP compared to no doses among women with at least two qualifying ANC visits, these exposures were a poor proxy for health care worker behavior. In addition to ANC attendance, individual-level factors associated with having received a full-course of IPTp included living in a rural residence, being less knowledgeable about the safety of SP use during pregnancy, having less education, living further from the ANC clinic, and the respondent being the household member who decides if SP will be used during pregnancy. The findings across the various studies, including ours, are not consistent with regard to these potential influences on receipt of IPTp [10], [11], [12], [14].

Similar to our findings, the primary reasons for not completing a full 2-dose course of IPTp among women who had received only 1 dose included not being given IPTp from the ANC and lack of awareness about the 2-dose schedule [36]. The lack of individual-level factors strongly associated with use of IPTp-SP in the literature, combined with our finding of the importance of having been offered SP during an ANC visit, suggest that factors related to health-care provider behavior are more influential in the uptake of IPTp than most measurable individual-level factors. Evaluations of health care provider practices regarding administration of IPTp are needed to measure the extent of the problem and develop targeted interventions at improving access to IPTp-SP during ANC visits. However, in light of the growing drug resistance to SP [37], [38], its continued use for IPTp may eventually be replaced by more effective alternative drugs which may require alternative dosing schedules.

This study is potentially subject to the following types of bias and limitations. Women who know IPTp is desirable and recommended may be relatively more likely to falsely report taking SP for prevention when they did not. However, based on the very high concordance among self-reported SP and SP recorded on the ANC card, the magnitude of this sort of social desirability bias is likely to be minimal. Additionally, we did not have information on medical contraindications to SP, such as history of sulfa drug allergies, or daily use of cotrimoxazole for the prevention of HIV-associated infections. If these factors were highly prevalent in our populations, misclassification would lead to an overestimate of the prevalence of missed opportunities, and the prevalence of appropriate SP use would be underestimated. Furthermore, suspected malaria in this area is treated presumptively, despite information which suggests relatively few cases which present with malaria-like symptoms actually have clinical malaria [39], [40], [41], [42]. If a large proportion of the women misclassified their reason for using SP, then our measure of IPTp could be biased in either direction. Lastly, we were unable to evaluate why health care providers failed to deliver IPTp for apparently eligible women.

The strengths of this study included use of a population-based random sample, validation of self-reported use of SP and ANC visits through an assessment of the ANC cards, and use of several visual aids throughout the interview to reduce recall bias. The visual aids included a pregnancy history calendar in which each pregnancy was mapped out over time on the calendar to record episodes of self-reported malaria, any use of SP or other antimalarials during pregnancy, and ANC visits, and photographs of commercial packaging for the most common formulations of SP available in the area, and the corresponding tablets.

In summary, we evaluated the level and determinants of use of IPTp during pregnancy. Our results indicate that few women in the Jinja district of Uganda were receiving the full 2-dose course of IPTp according to the recommendations, and that women are willing to take preventive SP in pregnancy if it is provided during an ANC visit. Missed opportunities to administer SP during ANC were common in our study, as was administration of SP in a manner that is not directed by the Ugandan guidelines, suggesting provider level improvements are needed. Improving healthcare providers' knowledge about the proper use and administration of SP may be an effective intervention to improve uptake, as well as simplifying the guidelines to provide IPTp-SP at each scheduled ANC visit after quickening.
